# The Homeopathic Preparation Neurexan® vs. Valerian for the Treatment of Insomnia: An Observational Study

**DOI:** 10.1100/tsw.2008.61

**Published:** 2008-04-20

**Authors:** Rainer Waldschütz, Peter Klein

**Affiliations:** ^1^ Hadwigstrasse 24, D - 78224 Singen, Germany; ^2^ d.s.h. statistical services GmbH Bahnhofstrasse 20, D - 85296 Rohrbach, Germany

**Keywords:** complementary medicine, sleep maintenance, sleep latency, sleep duration, homeopathy, tolerance

## Abstract

Insomnia is prevalent and complementary therapies are common, but data are lacking on the effectiveness and tolerability of preparations beyond valerian. Here we report on an open-label, prospective cohort study in 89 German centers offering both conventional and complementary therapies. Subjects received the homeopathic preparation Neurexan^®^ or valerian for 28 days. Doses were at physicians' judgments. Sleep duration and latency were evaluated based on patients' sleep diaries over 14 days; sleep quality was evaluated at 28 ± 1 days. A total of 409 subjects were enrolled. The groups were balanced at baseline for age, sex, weight, and sleep disturbances. At day 14, both groups reported improved sleep latency and duration; latency was reduced from baseline by 37.3 ± 36.3 min with Neurexan and by 38.2 ± 38.5 min with valerian. The duration of sleep increased by 2.2 (±1.6) h in the Neurexan group and by 2.0 (±1.5) h in the valerian group. Differences between the groups in improvement on sleep duration were significantly in favor of Neurexan therapy at days 8, 12, and 14. At day 28, quality of sleep was improved in both groups with no significant differences between the treatments. Significantly more patients reported lack of daytime fatigue with Neurexan than with valerian therapies (49% vs. 32%; *p* < 0.05 for the comparison). For patients favorable towards a CAM-based therapy, Neurexan might be an effective and well-tolerated alternative to conventional valerian-based therapies for the treatment of mild to moderate insomnia.

## INTRODUCTION

Insomnia is a highly prevalent condition; depending on the definition and methodology, surveys show that between 26% and 34% of the general population suffers from frequent sleeplessness[1,2]. The annual cost of insomnia to society in the U.S. has recently been estimated at between 92.5 and 107.5 billion U.S. dollars[3]. Many of the currently available agents used to treat insomnia, including antidepressants and nonbenzodiazepine hypnotics, have not consistently demonstrated effectiveness in promoting sleep maintenance. Furthermore, the benzodiazepines with established sleep maintenance efficacy are associated with side effects, such as "hangover" and the risk of developing tolerance and/or dependence[4].

Dissatisfaction with conventional medications is the major reason for the popularity of complementary and alternative medicine (CAM) in the U.S. and Europe[5,6], although CAM practices have a long history in countries such as China and Japan[7]. Among popular CAM treatments for insomnia are herbal preparations based on valerian. This has been used for centuries by the Greeks, Romans, Chinese, American Indians, and Europeans. Valerian root extract is used for treatment of insomnia at doses between 300 and 600 mg[8], and the dried root is used at 2–3 g, usually soaked in hot water[9]. Valerian extract was approved by the U.S. Commission E in 1985 for states of unrest and nervous sleep disturbance[10].

Studies in a range of settings, including randomized clinical trials, have demonstrated the efficacy of valerian[11,12,13,14]. It binds the same receptors as benzodiazepines[15] and preparations of valerian have been shown to be similarly effective as the benzodiazepam oxazepam in the treatment of nonorganic insomnia in a randomized, double-blind, comparative clinical study[16].

However, there are few data on the effectiveness of many CAM therapies beyond valerian in the treatment of insomnia. The current study investigated the homeopathic formulation Neurexan® (Heel GmbH, Baden-Baden, Germany). Neurexan is an over-the-counter (OTC) preparation based on highly diluted plant extracts, including valerianate of zinc. The components of Neurexan, shown in Table 1, are listed in the German Homeopathic Pharmacopoeia. In accordance with the principles of homotoxicology[17], most of the components are present at lesser dilutions than those commonly used in homeopathic preparations: $10^{-2}$ – $10^{-4}$ rather than the much higher dilutions typical in homeopathy. Neurexan is recommended to treat mild sleep disturbances and agitated conditions[18].

**TABLE 1**
**Components of Neurexan and Their Dilutions**

| Component | Common Name | Dilution | mg in Each Tablet |
|---|---|---|---|
| *Passiflora incarnata* | White sarsaparilla | D2 | 0.6 |
| *Avena sativa* | Common oats | D2 | 0.6 |
| *Coffea arabica* | Coffee tree | D12 | 0.6 |
| *Zincum isovalerianicum* | Valerianate of zinc | D4 | 0.6 |

The objective of the study was to assess the noninferiority of therapy with Neurexan compared with valerian therapy used at recommended doses during a period of 4 weeks, in patients with mild to moderate sleep onset and/or sleep maintenance insomnias. Complementary and alternative medications are used by a very wide range of individuals and in order to capture this variety, we chose an observational study design.

## METHODS

### Study Design

This was an open-label, prospective cohort study conducted in 89 German centers. Participating centers included practices offering both conventional therapy and CAM practices.

Inclusion criteria were age 18–75 years and a verified condition of mild to moderate sleep onset and/or sleep maintenance insomnias (sleep latency, low sleep quality, frequent nocturnal awakenings, sleep-associated impaired quality of life) diagnosed no longer than 4 weeks prior to enrollment. The conditions could be newly diagnosed or recurring. A minimum of three nights of insomnias a week was necessary, and the sleep disturbances were to have a significant negative impact on subjects' social and professional lives. Exclusion criteria were the presence of concomitant diseases and intolerance to any of the study medications or their components.

## Treatments

Patients were treated with Neurexan or valerian preparations for 28 days. There was no placebo group. To reflect everyday clinical CAM practice for insomnia, the choice of a commercial variety of valerian therapy was at the physicians' discretion. Doses were according to prescribers' judgments and were not stipulated in the study protocol. All patients were informed about the background and purpose of the study, which was conducted in full compliance with the principles of the Declaration of Helsinki[19] and with the German recommendations for the planning, execution, and evaluation of observational studies (Bundesanzeiger Federal Gazette No. 299 of December 04, 1998).

## Evaluations

Patients were examined at 14 ± 1 days and at the end of study at 28 ± 1 days. The effectiveness of the therapeutic regimens on subjective experiences of sleep latency and duration was evaluated as the difference between baseline and daily values, with the final analysis based on the data on day 14. These evaluations were based on patients' sleep diaries that recorded sleep latency (time to onset of sleep in minutes) and sleep duration (as hours slept during the night). The longer-term effects of therapy on quality of sleep were analyzed in a separate analysis as the difference between baseline values and the values on day 28.The assessment was based on a series of variables related to nocturnal and diurnal well-being. Change from baseline to the end of study at day 28 was graded on a scale from 0 to 3, where 0 indicates asymptomatic, 1 mild, 2 moderate, and 3 severe symptoms. For nocturnal effects, the summary score of the following sleep-related variables was calculated: frequent nocturnal awakenings, extended periods of wakefulness, agitated sleep, nervousness, recurring thought patterns, tossing, and turning. Diurnal residual effects were assessed on the variables fatigue, generally feeling unwell, lack of concentration, distress, emotional suffering, muscular aches, and anxiety related to the coming night.

Further, at the end of the study, an assessment of the overall effects of the therapies was done by the physician based on patient feedback. This assessment was done for the variables time to first sign of improvement, overall effectiveness of therapies, and the overall symptomatic change from the beginning of therapy to the time of evaluation. The percentages of patients who were asymptomatic at the end of therapy were also recorded.

## Statistical Methods

Summary statistics were calculated using absolute and percent number of ratings. Differences in baseline characteristics between treatment groups were adjusted for by propensity analysis as appropriate. Between-group differences were evaluated with the Cochran-Mantel-Haenszel test. Statistical comparisons were conducted with ANOVA and Fischer's exact test, as appropriate. For all comparisons, two-sided 95% confidence limits were calculated.

The aim of the study was to show noninferiority of Neurexan to valerian therapy and for this purpose, the effectiveness variables were analyzed in the per-protocol (PP) population. An additional intention-to-treat (ITT) analysis was performed to evaluate the robustness of the results.

Noninferiority was calculated based on the confidence limits for the differences in effectiveness between treatments. A noninferiority analysis was carried out for each of the individual symptoms for which treatment effect was assessed numerically as described above, as well as for the summary score of all variables. The borders of noninferiority were defined according to the investigators' judgment of clinically relevant differences between the medications. To fulfill the requirement of noninferiority, the limit for the lower boundary of the 95% confidence interval for the differences between the treatment groups was set to 10% of the maximal score ranges. The noninferiority analysis was conducted on the individual variables and on the summary score of all variables. However, there was no prespecified null hypothesis in this pilot study and all conclusions were of an exploratory nature.

Tolerability data were recorded as adverse events. In addition, the physician in dialogue assessed the overall tolerability of the treatment regimens with the patient as excellent, good, moderate, or poor. Compliance was rated by the physician on a similar rating scale from very good, good, moderate, to inadequate.

The study was conducted in 89 centers between August 25, 2004 and May 20, 2005. A total of 409 subjects aged 18–82 years were enrolled; 197 received Neurexan and 212 received valerian preparations.

## RESULTS

### Patient Population

Of the ITT population, 41 subjects in the Neurexan group and 48 subjects on valerian did not adhere to the protocol and were excluded from the PP population in the effectiveness evaluation. Thus, the PP population comprised 156 Neurexan subjects and 164 subjects receiving valerian. The mean treatment period (PP population) was 28 days in both treatment groups.

All patients in the Neurexan group received the regular dose of one to three tablets. In 22% of the population, additional tablets were taken at bedtime as indicated in the prescriber information. Most valerian doses in the control group ranged from 441.45 mg (Baldriparan®Stark capsules) to 600 mg (Sedonium 2 × 300-mg capsules). Other frequently used preparations were BALDRIAN-ratiopharm (450-mg capsules) and Euvegal®Balance (500-mg capsules). A small number of patients (6%) in the valerian group received a relatively low dose (Baldrian-dispert, 2 × 125-mg capsules).

The treatment groups were balanced at baseline for age, sex, weight, and the manifestations of the sleep disturbances (Table 2). Mean age was 50 years and there was a markedly greater percentage of women than men in both treatment groups; more than two-thirds of subjects in both groups were women. About half of subjects suffered from difficulties both in falling asleep and in maintaining an undisturbed sleep throughout the night (Table 2). At baseline, the mean sleep (estimated) latency in both groups was around 1 h and the mean number of hours slept in both groups was 4.7. The sleep disturbances were associated with reduced quality of life in about two-fifths of subjects in both treatment groups, with no significant differences between the groups.

### Therapeutic Effectiveness

The analysis of the therapeutic effectiveness after 14 days' treatment showed improvements from baseline in both sleep latency and in the duration of sleep with both therapies. The improvements in sleep latency were of similar magnitude in both groups; a reduction by 37.3 (±36.3) min with Neurexan therapy and 38.2 (±38.5) min with valerian treatment (between-group differences not significant). The duration of sleep (Fig. 1) increased by 2.2 (±1.6) h in the Neurexan group and by 2.0 (±1.5) h in the valerian group. The differences in sleep duration improvement between the groups (Fig. 1) were significantly in favor of Neurexan therapy at days 8, 12, and 14.

**TABLE 2**
**Demographic Criteria of the Subjects at Baseline (PP Population)***

| | Neurexan | Valerian |
|---|---|---|
| n | 156 | 164 |
| Age mean (SD) | 50.5 ± 14.1 | 50.1 ± 14.7 |
| Women n (%) | 120 (77%) | 112 (68%) |
| Weight kg (SD) | 73.7 ± 14.5 | 73.7 ± 13.2 |
| Average sleep latency in minutes (SD) | 59.8 ± 41.8 | 62.7 ± 41.7 |
| Average duration of sleep in hours (SD) | 4.7 ± 1.5 | 4.7 ± 1.3 |
| Manifestation of sleep disturbance n (%) | | |
| Acute insomnia and frequent awakenings | 88 (56%) | 100 (61%) |
| Low sleep quality | 79 (51%) | 92 (56%) |
| Reduced quality of life | 59 (38%) | 69 (42%) |
| Frequent awakenings | 44 (28%) | 33 (20%) |
| Early awakenings | 36 (23%) | 30 (18%) |
| Acute insomnia | 22 (14%) | 28 (17%) |
| Frequency of disturbances n (%) | | |
| Nightly | 65 (42%) | 50 (30%) |
| Five to six nights per week | 30 (19%) | 45 (27%) |
| Four nights per week | 37 (24%) | 38 (23%) |
| Three nights per week | 23 (15%) | 29 (18%) |
| Summary score nocturnal variables mean (SD) | 10.5 ± 3.7 | 11.0 ± 3.6 |
| Summary score diurnal variables mean (SD) | 10.7 ± 4.3 | 11.8 ± 4.2 |

* The differences between the treatment groups were not statistically significant.

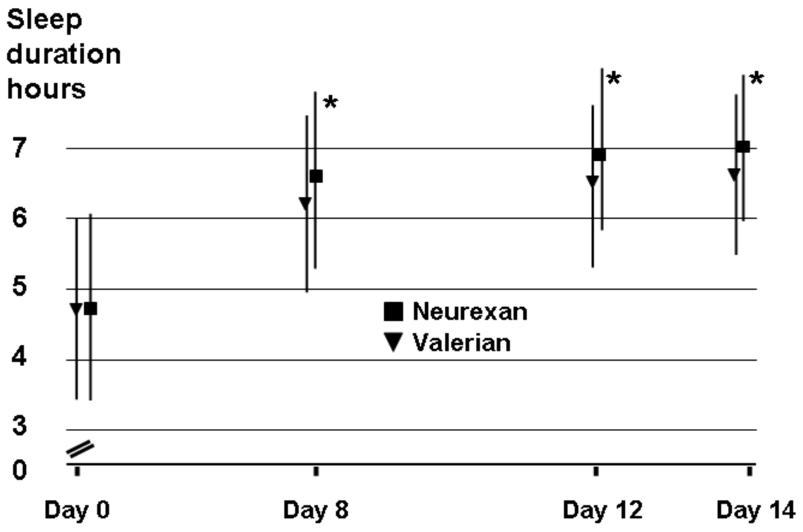

**FIGURE 1.** Mean estimated duration of sleep (hours) at time points with changes from baseline differed significantly between treatments (indicated by asterisks). Differences were in favor of Neurexan. Changes from baseline were significant for both groups. Lines indicate SD.

The analysis of the quality of sleep after 4 weeks of treatment also showed statistically significant improvements from baseline in the summary score of nocturnal as well as diurnal variables in both treatment groups. The mean nocturnal score was reduced by 7.5 (±3.5) units with Neurexan compared with a 7.3 (±4.1) reduction with valerian therapy. The mean diurnal score was reduced by 7.0 (±4.0) units with Neurexan vs. 7.2 (±4.2) with valerian therapy. There were no significant differences between the groups in effects on sleep quality.

The effects were consistent for all nocturnal variables in both groups. A noninferiority analysis of the differences between the two treatment groups is shown in Fig. 2. The differences in effects on the individual variables trended to favor Neurexan, although the differences did not reach significance. The lower boundaries of the 95% confidence intervals did not cross the boundary of –0.3 units, a result that supported the noninferiority of Neurexan on all individual variables.

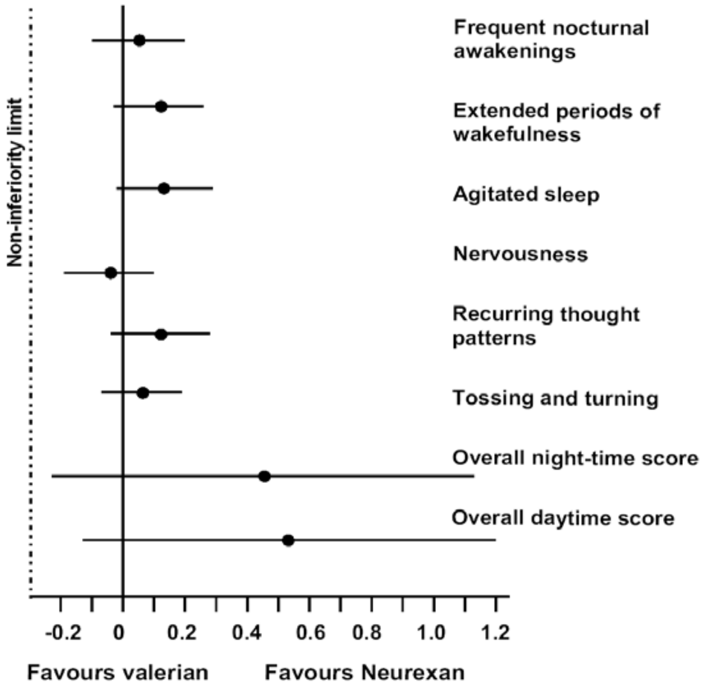

**FIGURE 2**. Mean differences and 95% confidence intervals for overall night-time and daytime therapeutic effectiveness between the two treatments, and for the components of the night-time score. Positive values favor Neurexan. The dotted line indicates the border for noninferiority of Neurexan to valerian therapy.

The noninferiority of the homeopathic treatment to valerian therapy was also demonstrated for the summary scores of night-time and daytime variables, respectively (Fig. 2).

A significant difference between the treatment group conditions was seen for daytime fatigue. Although a majority of subjects reported improvement in daytime fatigue in both treatment groups after 4 weeks of medication, 49% of subjects receiving Neurexan reported no daytime fatigue compared with 32% of subjects in the valerian group (Fig. 3; $p < 0.05$ for the differences). The majority of subjects on valerian reported mild fatigue at the end of the study.

In addition to the evaluations on individual variables and their summary score, treatment effectiveness was evaluated on the time of first signs of improvement, overall effectiveness, and overall symptomatic change since the beginning of therapy. Both therapies showed similar effectiveness on these evaluations.

The time to improvement was mostly in the range of 3–7 days. Of subjects in the Neurexan group, 65% reported benefits within this time period, compared with 59% of subjects in the valerian group. The overall

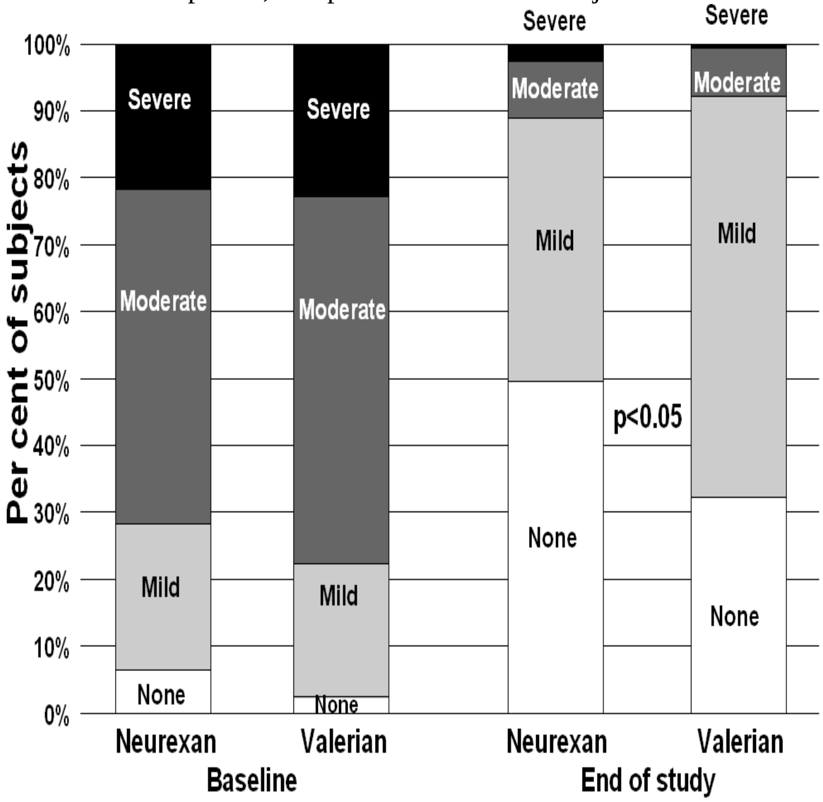

**FIGURE 3.** Daytime fatigue at baseline and end of study. The reduced amount of fatigue in the Neurexan group (n = 153) as compared to the valerian group (n = 164) at the end of the treatment period was statistically significant.

effectiveness was rated as “very high” or “high” in 86% of cases in the Neurexan group and in 85% on the control group. Similar high scores were observed for the overall symptomatic change from the beginning of therapy; 83% of patients in the Neurexan group and 84% in the control group rated their condition as “much improved” or “moderately improved”. Although there was a trend towards shorter time to improvement and more subjects reporting the highest degrees of effectiveness with the homeopathic therapy, the differences between treatment groups were not significant.

Both treatments reduced the number of subjects on sick leave due to insomnia. At the beginning of therapy, 18 subjects (12%) in the Neurexan group and 13 subjects (8%) in the valerian group were considered unable to work; at the end of the study period, the corresponding numbers were three (2%) and one (0.6%) patients in the two groups.

## Tolerance and Compliance

Both therapies were well tolerated, with only one adverse event occurring, i.e., a case of mild caffeine intolerance associated with Neurexan after 9 days of treatment. On overall tolerance, the evaluation “excellent” was given by 90% of subjects treated with Neurexan and 89% of subjects treated with valerian preparations.

A slight reduction in mean blood pressure was observed in both groups during the course of the therapy, with no differences between treatments. No other physiological changes were observed during the study.

Compliance rates were high with both therapeutic regimens. Physicians rated compliance as “very good” in 64% of Neurexan subjects and in 61% of valerian subjects. Inadequate compliance was reported for only two subjects (1%) in the Neurexan group and one subject (0.6%) in the control group. No differences in compliance ratings were observed between the therapeutic regimens.

## DISCUSSION

Many CAM strategies are used for sleep therapy[20], but very little research has been conducted on the effectiveness of insomnia and the quality of sleep. The present pilot study focused on a homeopathic preparation, Neurexan, which was compared with herbal valerian therapy over 14 and 28 days, with no placebo group included. The results indicate that this homeopathic therapy is an effective and well-tolerated alternative to conventional valerian therapies in this group of subjects with mild to moderate insomnia. The effects of treatments on sleep latency and duration at day 14 were highly similar in both groups. At day 28, sleep quality and daytime well-being improved to a similar degree with both treatments. An analysis showed the Neurexan regimen to be noninferior to valerian on all variables assessed.

The study was not designed to show superiority of Neurexan therapy, but it was notable that the greater effect on sleep duration at days 8, 12, and 14 with Neurexan than with valerian therapy were statistically significant. Improved sleep maintenance is not consistently achieved with many conventional therapies, such as antidepressants and nonbenzodiazepine hypnotics, and effective medications in the benzodiazepine class may cause side effects and intolerance[4]. At day 28, there was less daytime fatigue with the homeopathic treatment than with the valerian-based therapy.

The mechanisms behind the therapeutic benefits on sleep remain to be understood. Valerian has been used throughout millennia to treat insomnia and there are reports that the active ingredients have similar biological activities to benzodiazepam sedatives[15]. There are insufficient scientific data on Neurexan, as on many CAM preparations, and the current pilot study highlights the need both for large-scale clinical trials and for controlled laboratory experiments to build a scientific foundation for a mechanism of action. Valerian is a component of Neurexan (as valerianate of zinc), but this fact is unlikely, in itself, to account for the similar effects with the two regimens. The principles of homeopathy and homotoxicology rely on using very small doses of active substances to stimulate the body’s own protective mechanisms. Dosages of valerian root extract for treatment of insomnia range from 300 to 600 mg, which are concentrations several orders of magnitude higher that those found in Neurexan (Table 1). Thus, the effect of the valerian component of Neurexan is most probably due to a different mechanism of action to that of the herbal preparations.

Both therapies were very well tolerated. For Neurexan, this is in accordance with what is generally reported from homeopathic preparations. Valerian is also consistently well tolerated; reported side effects are headache, dizziness hangover, paradoxical stimulation, restlessness, and cardiac disturbances[21]. A potential worry is that valerian may potentiate the sedative effects of barbiturates, anesthetics, and other central nervous system depressants, and there might be a risk of withdrawal symptoms similar to those occurring with benzodiazepine use from long-term use of valerian therapy at high doses[22,23]. No such phenomena were observed during the current 28-day investigation, however.

This was a comparatively small study and some shortcomings are inevitable. First, there was no placebo group and thus the degree of placebo effect in both groups could not be assessed. However, in the case of valerian, a number of studies and controlled trials have reported benefits on sleep latency and maintenance[16]. As the current study was designed to compare the effectiveness of the therapies at the dosages and regimens used in clinical practice, the relevant end points were improved sleep as experienced by the patients, regardless of the possible contribution of a placebo component.

Second, this was an observational cohort study and patients were not randomized. The range of patients who opt for homeopathic therapies is very broad and treatment is highly individualized. The design of the study was determined by the need to include as wide a range of such patients as possible. The risk with a nonrandomized approach is that the patient groups may differ at baseline, which could introduce selection bias and reduce the validity of the conclusions[24]. However, in the current study, the differences between the groups at baseline were not significant, with symptoms in the Neurexan group very slightly and nonsignificantly milder than in the valerian group. Further, the fact that none of the preparations were from the field of conventional medicine argues for similar (patient and physician) expectations in both treatment groups, which would reduce the possibility of bias.

A further weakness was the relatively short duration of the study. The effects on insomnia over longer time periods, as well as the possible persistence of benefits after a washout, would be interesting to assess.

The definition of noninferiority can be discussed. No previous studies have validated limits for noninferiority using the variables and therapeutic regimens of the current study. Thus, the borders of noninferiority were defined according to the investigators' judgment of clinically relevant differences between the medications. This introduces an element of arbitrariness; however, most variables tended to favor the Neurexan group, which supports the conclusion of noninferiority.

In summary, the current observational study indicates that for patients favorable towards a CAM-based therapy, Neurexan might be an effective and well-tolerated alternative to conventional valerian-based therapies for the treatment of mild to moderate insomnia. The results suggest greater short-term effects with Neurexan on sleep duration and on daytime fatigue after 1 month of treatment. These findings encourage further clinical assessments in this and similar areas of natural medicine.
